# Prognostic value of left ventricular hypertrophy assessed with ^15^O-water positron emission tomography

**DOI:** 10.1093/ehjimp/qyag121

**Published:** 2026-07-03

**Authors:** Jonathan Sigfridsson, Patrik Svanström, Tanja Kero, Hendrik J Harms, Jonny Nordström, Mark Lubberink, Jens Sörensen

**Affiliations:** Molecular Imaging and Medical Physics, Department of Surgical Sciences, Uppsala University, Uppsala, Sweden; Molecular Imaging and Medical Physics, Department of Surgical Sciences, Uppsala University, Uppsala, Sweden; Molecular Imaging and Medical Physics, Department of Surgical Sciences, Uppsala University, Uppsala, Sweden; Molecular Imaging and Medical Physics, Department of Surgical Sciences, Uppsala University, Uppsala, Sweden; Molecular Imaging and Medical Physics, Department of Surgical Sciences, Uppsala University, Uppsala, Sweden; Uppsala/Gävleborg County, Centre for Research and Development, Gävle, Sweden; Molecular Imaging and Medical Physics, Department of Surgical Sciences, Uppsala University, Uppsala, Sweden; Molecular Imaging and Medical Physics, Department of Surgical Sciences, Uppsala University, Uppsala, Sweden

**Keywords:** coronary artery disease, heart failure, left ventricular hypertrophy, cardiac PET, prognosis

## Abstract

**Aims:**

Left ventricular (LV) hypertrophy is associated with adverse cardiac outcomes. LV mass index (LVMi) and interventricular septal wall thickness (WT) can be derived from ^15^O-water positron emission tomography (PET), but their prognostic value is unknown. We aimed to assess the prognostic value and test–retest repeatability of PET-derived LV hypertrophy metrics in patients evaluated for suspected coronary artery disease (CAD).

**Methods and results:**

We retrospectively included 783 consecutive patients referred for rest-stress PET. LVMi and WT were determined on non-ECG-gated resting ^15^O-water PET scans. Subjects were stratified by previous heart failure (HF) diagnosis and patients with known hypertrophic/infiltrative cardiomyopathy were excluded. The primary outcome was major adverse cardiac events (MACE: cardiovascular death or acute HF). The prognostic value of LV hypertrophy measurements was evaluated using Cox hazard ratios (HR [95% confidence interval]). Test–retest repeatability was evaluated in prospective data from 21 patients with repeated PET scans.

Patients with known HF but no hypertrophic/infiltrative cardiomyopathy (18%) had significantly higher LVMi (*P* < 0.0001) and WT (*P* = 0.0015) compared to subjects without HF (82%). Among patients without HF, 30 experienced MACE during a median 3.8 year follow up. Both LVMi (HR 1.04 [1.02–1.06] per g/m^2^, *P* < 0.0001) and WT (HR 1.36 [1.10–1.68] per mm, *P* = 0.0045) independently predicted MACE after adjustment for sex, age and myocardial flow reserve. Test–retest repeatability was high, with no significant bias.

**Conclusion:**

PET LV hypertrophy metrics can be robustly quantified from routine ^15^O-water PET and provide prognostic information beyond myocardial perfusion.

## Graphical Abstract

**Graphical Abstract:**
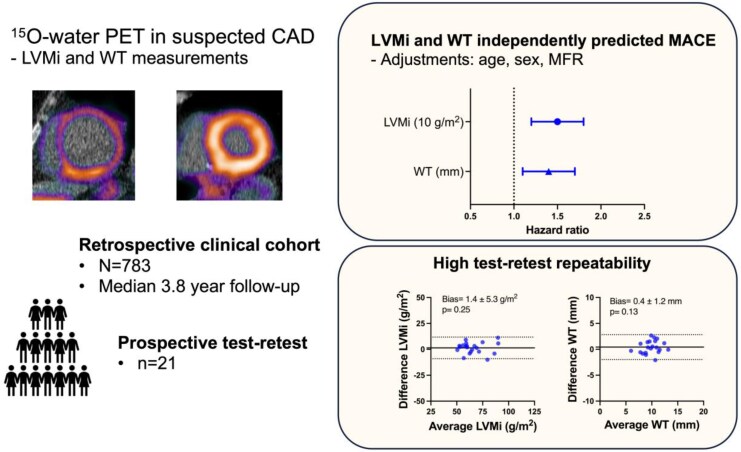
LVMi and WT were determined on ^15^O-water perfusable tissue fraction images in 783 patients from a retrospective clinical cohort and 21 patients in a prospective test–retest study. PET-based LVMi and WT were predictive of major adverse cardiac events and both metrics were highly reproducible. Abbreviations: CAD, coronary artery disease; LVMi, left ventricular mass index; MFR, myocardial flow reserve; PET, positron emission tomography; WT, interventricular septal wall thickness.

## Introduction

Left ventricular (LV) hypertrophy is a powerful predictor of mortality and adverse cardiac outcomes across multiple cardiovascular conditions, including coronary artery disease (CAD).^1–3^ Early detection and regression of LV hypertrophy improve prognosis,^4,5^ underscoring the need for accurate and reproducible assessment. Two important LV hypertrophy metrics are LV mass and interventricular septal wall thickness (WT). Echocardiography is widely used for assessment of LV hypertrophy, but has known limitations for LV mass quantification due to geometric assumptions and operator dependency.^6^ Cardiac magnetic resonance (CMR) is the reference standard for assessment of both LV mass and WT but has lower availability and is associated with contraindications related to metal implants and claustrophobia.

LV hypertrophy is associated with reduced myocardial perfusion and coronary microvascular dysfunction.^7,8^ This relationship suggests a potential clinical utility of simultaneously evaluating LV hypertrophy and myocardial perfusion using positron emission tomography (PET). PET with ^15^O-water is considered the reference method for quantification of myocardial perfusion owing to its excellent tracer characteristics and high accuracy for detecting haemodynamically significant CAD.^9–11^ Kinetic modelling of ^15^O-water PET allows for quantification of absolute myocardial blood flow (MBF) and among other parameters, the perfusable tissue fraction (PTF). The PTF is used to correct for partial volume effects and non-perfused tissue but corresponding parametric images provides high contrast information of the myocardial wall useful for LV myocardium delineation.^12,13^ One prior study showed that PTF images from ^15^O-water PET provided accurate measurements of LV mass and WT when compared with CMR.^14^ As this technique is fully automatic, combined LV hypertrophy measurements with absolute MBF quantification seem feasible and an interesting approach for clinical routine scanning.

To date, one previous publication has addressed the incremental predictive value of PET LV hypertrophy assessments where they found that LV mass from ^82^Rb PET gave no incremental prognostic information compared to myocardial perfusion measurements.^15^ However, no such study has been performed using ^15^O-water PET LV mass and moreover, the prognostic value of PET-based WT is thus far unknown for any tracer. Additionally, the test–retest reproducibility of ^15^O-water LV mass and WT has not been evaluated.

Consequently, we aimed to (i) assess the prognostic value of ^15^O-water PET-derived LV hypertrophy metrics in patients with suspected CAD, and (ii) determine their test-retest reproducibility.

## Methods

### Study population

The study was performed using data from Uppsala University Hospital in Sweden. First, we included retrospective data from consecutive patients who underwent clinical routine scanning with ^15^O-water PET between 2012 and 2022 due to suspected CAD. Patients residing outside Uppsala County were excluded due to limited availability of medical records. Secondly, to assess the reproducibility of PET measurements, a prospective cohort of patients who underwent controlled test–retest scanning with ^15^O-water PET was included. The patients performed the first scan as part of their routine evaluation for suspected CAD and were asked to repeat the scan later on the same day.

The requirement for written informed consent was waived in the clinical CAD cohort. All patients in the test–retest cohort provided written consent. The studies were conducted in accordance with the Declaration of Helsinki and approved by the Swedish Ethical Review Authority (Registration Number: clinical CAD cohort: 2022-05360; test–retest cohort: 2022-01114-01).

### PET imaging

The routine PET protocol consisted of a low-dose computed tomography (CT) scan followed by two 4 min dynamic PET acquisitions during rest and pharmacological stress. PET scanning started simultaneous with the injection of 400 MBq ^15^O-water as a controlled bolus (10 mL at 0.8 mL/s followed by 30 mL saline at 2.0 mL/s). Pharmacological stress was mainly induced with adenosine infusion (duration 6 min, starting 2 min pre ^15^O-water injection), using regadenoson only in case of moderate or severe asthma. Patients in the test–retest cohort repeated the scanning during the same day after an approximately 2-h waiting period. Only adenosine was used for vasodilation in the test–retest cohort.

In the CAD cohort, three different PET/CT scanners were used: a digital scanner (Discovery MI, GE HealthCare) or older scanners with bismuth germanate oxide (BGO) detectors (Discovery ST or Discovery IQ, GE HealthCare). Test–retest scanning was performed only on the digital PET/CT.

### PET image analysis

Image processing was performed using aQuant Research (MedTrace Pharma A/S, Hørsholm, Denmark). Arterial and venous blood input functions were derived from the dynamic images with cluster analysis as described elsewhere.^16^ Parametric images were generated, and global LV rest and stress MBF and PTF were quantified according to routine procedure.^17^ Global LV myocardial flow reserve (MFR) was calculated as stress divided by rest MBF.

**Figure 1:**
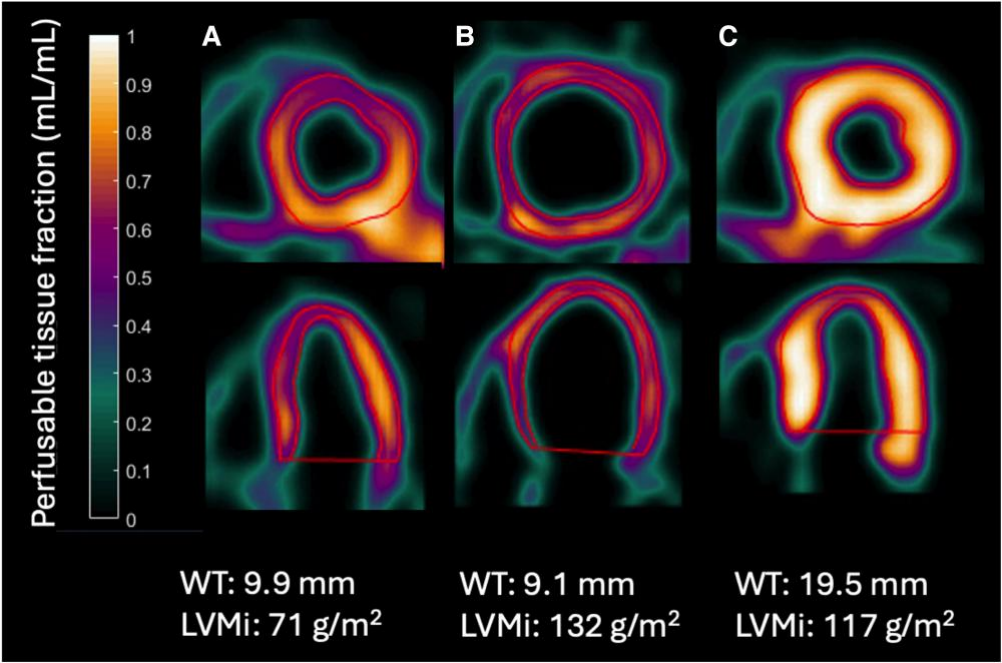
^15^O-water perfusable tissue fraction (PTF) images reoriented to cardiac short axis (upper panel) and horizontal long axis (lower panel) views showing patients with different cardiac geometry. Colour scale maximum is PTF = 1.0. (*A*) Male patient with normal PET findings and no known left ventricular hypertrophy. (*B*) Female patient with dilated cardiomyopathy. (*C*) male patient with cardiac amyloidosis and left ventricular hypertrophy confirmed by echocardiography. WT, interventricular septal wall thickness; LVMi, left ventricular mass index.

LV segmentation for LV mass and WT calculations was performed on parametric PTF images. The PTF images were transformed into short-axis orientation and the basal slice was defined where ≥50% of the myocardial circumference was present. Myocardial contours were automatically delineated using a relative PTF threshold of 67% applied in 10 degrees radial profiles, as previously validated.^14^ Manual corrections of the LV myocardium volumes of interest were carried out when required. Representative images of the LV delineation over different types of cardiac geometry are presented in *Figure 1*.

To determine LV mass, the total volume of voxels inside the segmented LV myocardium was multiplied by the density of myocardial tissue, fixed at 1.05 g/mL. LV mass was indexed to body surface area and denoted as LVMi. Measurement of WT was performed automatically on the average of five profiles at the border of the mid-ventricular anteroseptal and inferoseptal segments.

### Statistical analysis

Data were tested for normality and mean ± standard deviation (SD) or median and interquartile range were used to describe continuous variables. Categorical data were presented as counts with percentages. Group comparisons utilized *t*-tests, Kruskal–Wallis tests, Wilcoxon rank-sum tests, and Pearson’s chi-square tests, as appropriate. Standard least squares models were applied to study associations between PET scanner model and LV hypertrophy metrics.

Test–retest reproducibility was evaluated using Bland–Altman analysis in the controlled test–retest cohort. Coefficient of variation was defined as the standard deviation of the test-retest differences divided by the mean.

The prognostic value of PET LV hypertrophy metrics was examined with Cox proportional hazard models. Additionally, we performed Kaplan–Meier analyses with log-rank tests based on diagnostic cut-off values. Sex-specific cut-offs for LV hypertrophy were derived from previous CMR and echocardiography literature. For LVMi, CMR determined cut-offs based on a recent meta-analysis were used.^18^ For WT cut-offs, we utilized echocardiographic guidelines for cardiac chamber quantification.^19^ Subjects were stratified based on previous diagnosis of heart failure (HF), and patients with history of HF or hypertrophic or infiltrative cardiomyopathy were excluded from the survival analyses. Primary outcome was time to major adverse cardiac event (MACE) defined as a composite of cardiovascular death and hospitalization for acute HF.

Two-sided *P*-values <0.05 were considered statistically significant. Statistical calculations were performed in JMP 19 (SAS Institute Inc., Cary, NC, USA).

## Results

### Clinical CAD cohort characteristics

Patient characteristics and PET measurements in the clinical CAD cohort are presented in *Table 1*. In total, 886 consecutive patients were scanned during the inclusion period. Medical records were available for 783 patients (45% females). Of these, 149 patients had known HF, 8 had known hypertrophic cardiomyopathy, and 3 had cardiac amyloidosis. Five of the eight patients with hypertrophic cardiomyopathy and two of the three patients with cardiac amyloidosis had concomitant HF.

**Table 1:** Patient characteristics in the retrospective clinical coronary artery disease cohort

	All (*n* = 783)	Females (*n* = 356)	Males (*n* = 427)	*P*-value
Demographics				
Age (years)	68 [59–74]	67 [57–75]	68 [60–74]	0.4
Body-mass index (kg/m^2^)	27 [25–31]	28 [24–31]	27 [25–31]	0.9
Body-surface area (m^2^)	2.0 [1.8–2.1]	1.8 [1.7–1.9]	2.0 [1.9–2.2]	<0.0001
Clinical data				
Heart rate (beats/min)	68 [60–77]	71 [63–80]	66 [59–75]	<0.0001
Systolic blood pressure (mmHg)	131 [120–145]	132 [120–145]	131 [120–145]	0.5
Diastolic blood pressure (mmHg)	74 [67–82]	74 [67–82]	74 [67–83]	0.5
Heart failure (*n*)	149 (19%)	54 (15%)	95 (22%)	0.01
Hypertrophic cardiomyopathy (*n*)	8 (1%)	1 (<1%)	7 (2%)	N/A
Cardiac amyloidosis (n)	3 (<1%)	2 (<1%)	1 (<1%)	N/A
Diabetes (*n*)	206 (26%)	75 (21%)	131 (31%)	0.002
Hypertension (*n*)	513 (66%)	225 (63%)	288 (67%)	0.2
Hyperlipidaemia (*n*)	329 (42%)	133 (37%)	196 (46%)	0.02
Smoking (*n*)	400 (51%)	166 (47%)	234 (55%)	0.02
Dyspnoea (*n*)	194 (25%)	96 (27%)	98 (23%)	0.2
Chest pain (*n*)	530 (68%)	253 (71%)	277 (65%)	0.1
PET measurements				
Rest myocardial blood flow (mL/g/min)	1.0 [0.8–1.2]	1.1 [0.9–1.3]	0.8 [0.7–1.0]	<0.0001
Stress myocardial blood flow (mL/g/min)	2.8 [2.0–3.6]	3.4 [2.6–4.1]	2.4 [1.8–3.0]	<0.0001
Myocardial flow reserve	2.8 [2.2–3.5]	3.0 [2.3–3.6]	2.8 [2.0–3.4]	0.002
Myocardial flow reserve <2.5 (*n*)	269 (34%)	111 (31%)	158 (37%)	0.09
Left ventricular mass (g)	126 [105–153]	106 [93–122]	147 [126–170]	<0.0001
Left ventricular mass index (g/m^2^)	65 [56–76]	58 [53–65]	71 [63–81]	<0.0001
Interventricular septal wall thickness (mm)	10.1 [8.8–11.5]	9.3 [8.3–10.6]	10.7 [9.4–12.1]	<0.0001

Continuous data were non-normal and are presented as median and interquartile range. *P*-values are based on Wilcoxon’s two-sample tests and Pearson’s Chi-square tests for proportions

Due to similar technical PET detector characteristics, and few subjects (*n* = 6) scanned on the IQ PET/CT scanner, data was pooled as either derived from digital PET/CT (*n* = 615) or BGO PET/CT (*n* = 168). Standard least squares models studying the effect of scanner type on LV hypertrophy metrics were performed with adjustments for sex, age, body mass index, global LV MFR, known hypertension, hyperlipidaemia, diabetes, known HF, and hypertrophic/infiltrative cardiomyopathy. Patients scanned with the digital scanner had 5% larger adjusted LVMi (difference 3.7 [1.3–6.1] g/m^2^, *P* = 0.002) and 4% lower WT (difference −0.49 [−0.79; −0.20] mm, *P* = 0.001), compared to BGO PET/CT.

**Figure 2:**
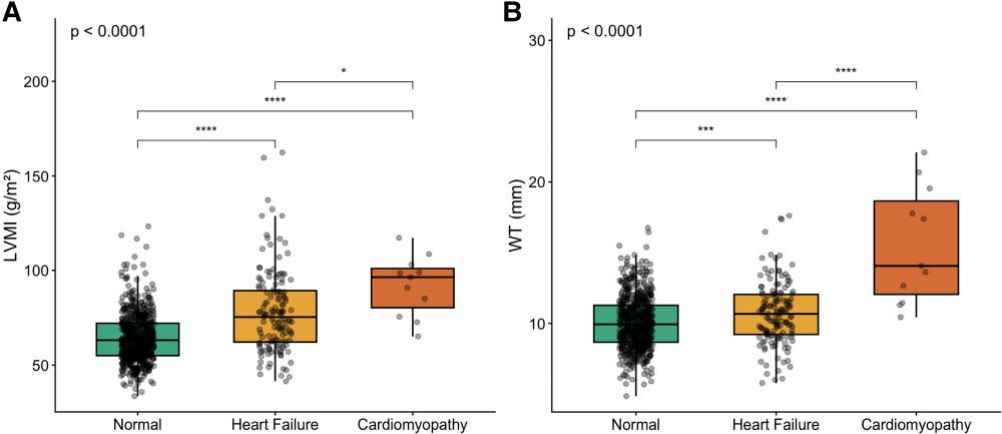
Boxplots showing the distribution of left ventricular mass index (LVMi) (*A*) and interventricular septal wall thickness (WT) (*B*) in patients without heart failure or hypertrophic/infiltrative cardiomyopathy, in heart failure patients, and in patients with hypertrophic/infiltrative cardiomyopathy. Boxes represent the interquartile range (IQR) with median values indicated, whiskers denote values within 1.5 × IQR, and individual data points are shown. Overall group differences were assessed using the Kruskal–Wallis test, with pairwise post-hoc comparisons performed with Wilcoxon rank-sum test and Benjamini–Hochberg correction for multiple testing. Statistical significance in the post-hoc tests is indicated by asterisks (*P < 0.05, **P < 0.01, ***P < 0.001, ****P < 0.0001).

In patients without hypertrophic or infiltrative cardiomyopathy, males had higher median LVMi (71 [62–80] vs. 58 [53–65] g/m^2^) and WT (10.7 [9.4–12.0] vs. 9.3 [8.3–10.6] mm) vs. females (both *P* < 0.0001). The 11 patients with hypertrophic or infiltrative cardiomyopathy (3 females) had higher LVMi (96 [76–103] vs. 64 [56–75] g/m^2^, *P* < 0.0001) and WT (14.1 [11.4–19.5] vs. 10.0 [8.8–11.4] mm, *P* < 0.0001). Subjects with a history of HF but no hypertrophic/infiltrative cardiomyopathy (*n* = 142 (52 females)) had 18% higher median LVMi (*P* < 0.0001) and 7% higher WT (*P* = 0.0015) vs. non-HF. Significant differences were found between patients without heart failure or hypertrophic/infiltrative cardiomyopathy, patients with heart failure, and patients with hypertrophic/infiltrative cardiomyopathy (*Figure 2*).

### Prognostic value of PET LV hypertrophy measurements

Among 630 patients (301 females) without known HF or hypertrophic/infiltrative cardiomyopathy, 30 patients (15 females) experienced MACE during 3.8 (IQR: 2.5–6.2) years follow-up.

Multivariate Cox models were performed with continuous LVMi and WT measurements, adjusting for sex, age and global LV MFR. LVMi (HR 1.04 [1.02–1.06] per g/m^2^, *P* < 0.0001) and WT (HR 1.36 [1.10–1.68] per mm, *P* = 0.0045) were independently predictive of MACE. In patients with known HF, using the same adjustments, LVMi was borderline significant (*P* = 0.046) and WT was not significant (*P* = 0.18). When adding both hypertrophy metrics to the same model in patients without HF, LVMi remained a significant predictor (*P* = 0.0045) while WT lost significance (*P* = 0.18). In all models, for both non-HF and HF patients, age and MFR withheld their significance, but sex did not.

**Figure 3:**
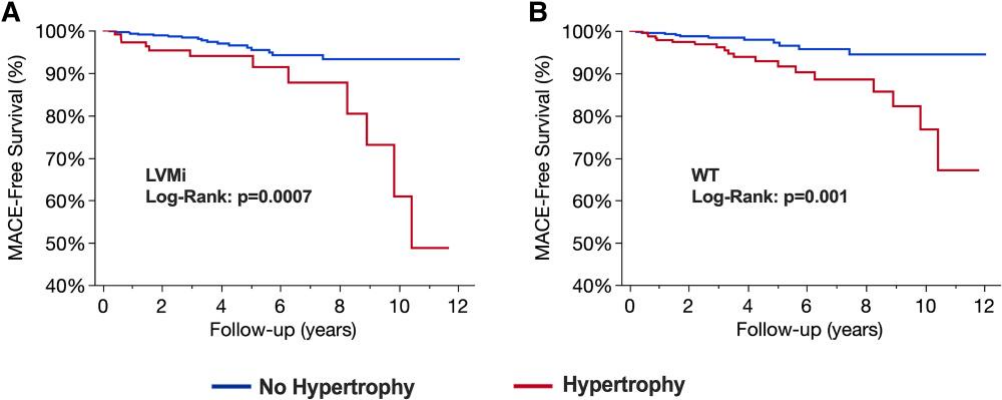
Kaplan–Meier survival analysis of left ventricular mass index (LVMi) (*A*) and interventricular septal wall thickness (WT) (*B*). Outcome was time to major adverse cardiac event (MACE) defined as a composite of acute heart failure or cardiovascular death. Patients with known heart failure and/or hypertrophic or infiltrative cardiomyopathy were excluded. Blue lines represent patients with low- or normal values and red lines represent left ventricular hypertrophy. Cut-offs were derived from the literature utilizing upper normal values from cardiovascular magnetic resonance imaging (LVMi) and echocardiography (WT). Sex-specific hypertrophy cut-offs were LVMi >65 g/m^2^ (female) and >83 g/m^2^ (male), and WT >10 mm (female) and >11 mm (male).

Additionally, we performed predictive analyses with CMR-based LVMi cut-offs (females: 65 g/m^2^, males: 83 g/m^2^) and echocardiographic WT cut-offs (females: 10 mm, males: 11 mm). Using Kaplan–Meier analyses with log-rank test, elevated LVMi (*P* = 0.0007) and WT (*P* = 0.001) were associated with higher event rates (*Figure 3*). In univariate Cox regression analyses, high LVMi (HR 3.32 [1.60–6.91], *P* = 0.0013) and WT (HR 3.25 [1.54–6.83], *P* = 0.0019) were, respectively, associated with increased hazard ratios. For patients with known HF neither elevated LVMi (*P* = 0.46) or WT (*P* = 0.67) were significant.

### Test–retest of PET LV hypertrophy measurements

In the prospective test–retest study, 21 patients were scanned twice. Of these, 19 underwent same-day scanning, while two repeated the scan after 35 and 41 days, respectively, due to logistic issues.

**Figure 4:**
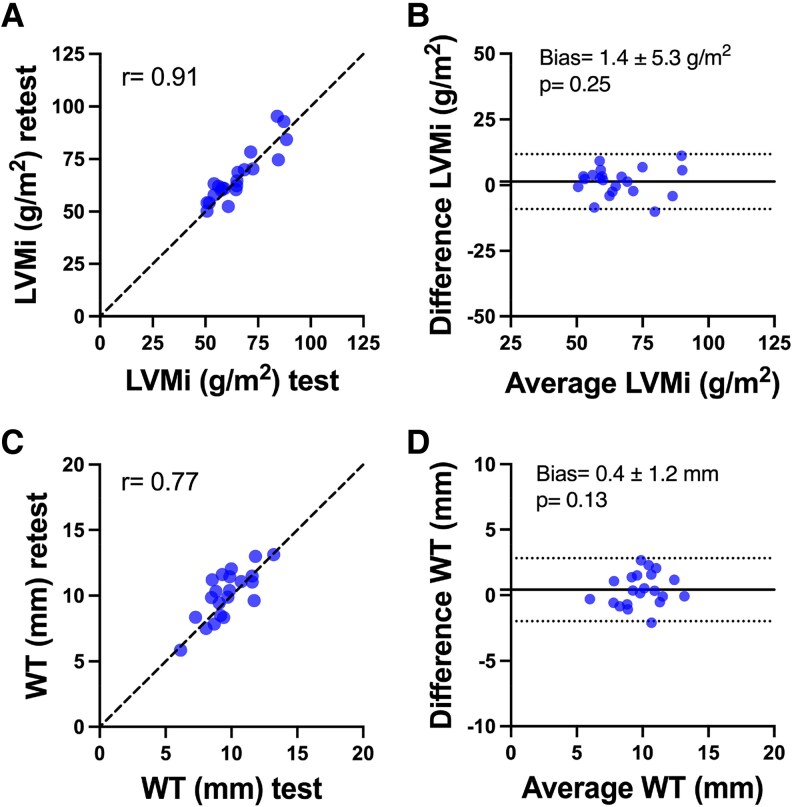
Test–retest of positron emission tomography left ventricular mass index (LVMi) and interventricular septal wall thickness (WT) in *n* = 21 patients. Dashed lines are lines of identity (*A*, *C*), dotted lines represent Bland–Altman 95% limits of agreement (*B*, *D*), and solid lines represent bias (*B*, *D*).

Test–retest repeatability results are shown in *Table 2* and *Figure 4*. LV hypertrophy measurements were highly reproducible with no significant bias in Bland–Altman analysis. Linear regression showed a very strong correlation for LVMi (*r* = 0.91), and a strong correlation for WT (*r* = 0.77) (both *P* < 0.0001). Repeatability coefficient (SD of difference × 1.96) was 10.4 g/m^2^ for LVMi and 2.4 mm for WT. Corresponding CV was 8% for LVMi and 12% for WT.

**Table 2:** Test–retest of positron emission tomography based left ventricular hypertrophy measurements

	Left ventricular mass index (g/m^2^)	Interventricular septal wall thickness (mm)
Mean test ± SD	65.2 ± 12.2	9.7 ± 1.7
Mean retest ± SD	66.6 ± 12.5	10.1 ± 1.9
Bias ± SD	1.4 ± 5.3	0.4 ± 1.2
LoA	−9.1 to 11.8	−1.98 to 2.82
CV (%)	8	12
*P*-value (*t*-test)	0.25	0.13
Pearsons *r*	0.91	0.77
*P*-value (correlation)	<0.0001	<0.0001

Bias ± standard deviation (SD), limits of agreement (LoA), and coefficient of variation (CV) was assessed with Bland–Altman plots. CV was defined as SD of the difference divided by the mean of the two measurements.

## Discussion

In this study, we demonstrate that LV hypertrophy metrics can be derived from routine ^15^O-water PET. Using a highly automated approach, LVMi and WT measurements could be achieved in all subjects. The method utilizes the same data as for regular myocardial perfusion quantification, avoiding the need for extra data collection and post-processing. Both LVMi and WT showed excellent test–retest reproducibility and predicted cardiovascular events in patients with suspected CAD, even after adjustment for MFR. These findings indicate that PET-based assessment of cardiac structure adds clinically relevant information to standard perfusion imaging. Although PET is typically performed later in the diagnostic process, when potential hypertrophy is often already diagnosed, there are occasions when diagnosing LV hypertrophy with PET could be important, such as in patients whose prior echocardiographic assessments were performed remotely in time. As indicated in *Figure 1*, inspection of routinely calculated parametric PTF images might provide visual aid in diagnosing LV hypertrophy and remodelling in ^15^O-water PET.

The high repeatability found in this study was in line with previous results. A controlled test-retest reproducibility of PET-based LV mass has previously been assessed with ECG-gated ^82^Rb in healthy controls, where a high agreement was seen.^20^ The foundation for the PET LV mass and WT calculation used in the current study was initially presented for ^11^C-acetate PET,^21^ for which the parametric images of K_1_ uptake rate were used for segmentation. The results showed that non-gated ^11^C-acetate PET LV mass and WT were highly accurate and reproducible. Furthermore, hypertrophy assessments using ^15^O-water PET based on PTF images were recently validated towards CMR and echocardiography, achieving high accuracies for detection of abnormal LV mass and WT.^14^ Altogether, this strengthens the theory that LV mass and WT calculations from PET is robust and can be performed with any perfusion tracer when kinetic modelling with formation of parametric images is applied. Noteworthy, however, is that the non-gated ^15^O-water method measures WT in a small region at the mid-ventricular level, chosen as it is least affected by motion and contraction. In case of myocardial scar in this region, the automated approach will result in a falsely low WT, while prolonged contractions will probably result in overestimations. Additionally, echocardiography and CMR can detect focal hypertrophy elsewhere in the myocardial wall, which is potentially quantifiable by an automated PET approach only when using ECG-gated images.

In accordance with earlier research, LV hypertrophy in this study was associated with adverse cardiac outcomes. Interestingly, the prognostic relevance was not seen as clearly in patients with already known HF. This suggests that other factors, such as poor hyperaemic MBF or reduced pump function drive the deterioration towards MACE in established HF. In the absence of a primary hypertrophic cardiomyopathy, elevated LV mass is primarily considered an indicator of chronically elevated loading in which hypertrophy development is a secondary phenomenon, countering increased wall tension. Increased LVMi is, as such, anticipated in subjects with chronic HF, which was also shown in this study with 18% higher mass in subjects with history of HF. Diagnosing increased LV mass in asymptomatic or anginal subjects without HF is a harbinger of future adverse events, providing an opportunity to address causative processes.

A previous study showed no incremental prognostic benefit for ^82^Rb-PET LVMi in addition to perfusion measurements,^15^ contrasting our findings where LV hypertrophy, and LVMi in particular, remained prognostic relative to MFR. This discrepancy might be due to several reasons: One explanation could be the difference in primary outcome, where the current study utilized a composite of cardiovascular death and acute HF, whereas the previous study targeted death and coronary events. Additionally, the few events that occurred during follow-up in our cohort limited the number of variables reasonable to use for adjusting the predictive Cox models and therefore, the models differed.

To the best of our knowledge, this is the first study to show the prognostic value of PET-based WT measurements. While the added value of assessing LV structure besides LV mass has been debated,^22,23^ most evidence suggests that it is relevant to also study other aspects of cardiac geometry.^4,24,25^ Increased WT is generally considered a hallmark of primary cardiomyopathies with hypertrophy, which is important to screen for. Furthermore, 2D-echocardiography, the primary imaging modality in most clinical scenarios, relies on geometric assumptions and requires an adequate acoustic window when performing LV mass calculations. This induces measurement variability and therefore, WT rather than LV mass is often used as the primary hypertrophy metric, additionally highlighting the relevance of reporting WT in between echocardiography follow-ups with other imaging modalities.

We defined LV hypertrophy using previously validated cut-offs from CMR^18^ and echocardiography^19^ and found that elevated LVMi and WT calculated with ^15^O-water PET predicted MACE. While prognostic value alone does not establish diagnostic equivalence, this indicates that established and clinically validated thresholds may have potential applicability in routine PET myocardial perfusion imaging for identification of LV hypertrophy. Furthermore, a previous study demonstrated high agreement between ^15^O-water and conventionally derived LV hypertrophy measurements.^14^ Use of standardized cut-offs could enhance integration with complementary imaging findings during multimodality diagnostic work-up and improve consistency. Nevertheless, for diagnostic and prognostic purposes, it would be relevant to determine PET specific normal ranges and cut-off values. Larger studies are warranted to determine such cut-offs for routine scanning and evaluate other aspects of cardiac hypertrophy and remodelling assessments with PET. Multicentric studies seem feasible for that purpose, taking scanner differences into account, as a small but significant difference between high-end and low-end PET scanners was found in the current study.

### Limitations

Limitations in this study include the relatively small number of events which means that a few outliers could have had large effects on the statistical analysis. The number of events were further reduced due to exclusion of patients with known HF. The choice to do so was to limit the effect of potential confounding factors. Given this, the multivariable models intentionally included a limited number of variables to avoid overfitting. Adjustments were restricted to age and sex as fundamental determinants of cardiovascular risk, together with myocardial perfusion reserve, a well-established prognostic marker in PET myocardial perfusion imaging. This approach allowed us to evaluate the prognostic value of PET-derived LV hypertrophy beyond key demographic factors and perfusion, while maintaining model stability. However, the multivariable analyses were thus not adjusted for other important risk factors and comorbidities associated with LV hypertrophy, such as hypertension, diabetes and obesity, which represents a limitation and should be considered when interpreting the results. Given the limited number of covariates included, the possibility of residual confounding cannot be excluded.

All PET examinations were performed in a single centre which might limit generalizability. PET with ^15^O-water is not yet a widespread technique for routine myocardial perfusion imaging and therefore, the proposed methods are not directly translatable to many sites in the world. However, the techniques presented could be applied to other more commonly used tracers with only minor alterations.

### Conclusion

Assessment of LV hypertrophy from routine ^15^O-water PET is feasible, reproducible, and provides prognostic information beyond myocardial perfusion. These metrics may enhance clinical risk assessment during PET evaluation of suspected CAD.

## Data Availability

The data underlying this article are available from the corresponding author upon reasonable request.

## Lead author biography

Jonathan Sigfridsson is a nuclear medicine technologist with an MSc and currently a PhD student at The Department of Surgical Sciences, Uppsala University, Uppsala, Sweden. His research focuses on cardiovascular molecular imaging, with particular interest in developing PET techniques for quantifying cardiac function and geometry.
